# Flow cytometry as a rapid analytical tool to determine physiological responses to changing O_2_ and iron concentration by *Magnetospirillum gryphiswaldense* strain MSR-1

**DOI:** 10.1038/s41598-017-13414-z

**Published:** 2017-10-13

**Authors:** Alfred Fernández-Castané, Hong Li, Owen R. T. Thomas, Tim W. Overton

**Affiliations:** 1 School of Chemical Engineering, College of Engineering and Physical Sciences, University of Birmingham, Edgbaston, Birmingham B15 2TT UK; 2 Institute for Microbiology and Infection, University of Birmingham, Edgbaston, Birmingham B15 2TT UK; 3 Present Address: School of Engineering and Applied Science, Aston University, Birmingham, B4 7ET UK

## Abstract

Magnetotactic bacteria (MTB) are a diverse group of bacteria that synthesise magnetosomes, magnetic membrane-bound nanoparticles that have a variety of diagnostic, clinical and biotechnological applications. We present the development of rapid methods using flow cytometry to characterize several aspects of the physiology of the commonly-used MTB *Magnetospirillum gryphiswaldense* MSR-1. Flow cytometry is an optical technique that rapidly measures characteristics of individual bacteria within a culture, thereby allowing determination of population heterogeneity and also permitting direct analysis of bacteria. Scatter measurements were used to measure and compare bacterial size, shape and morphology. Membrane permeability and polarization were measured using the dyes propidium iodide and bis-(1,3-dibutylbarbituric acid) trimethine oxonol to determine the viability and ‘health’ of bacteria. Dyes were also used to determine changes in concentration of intracellular free iron and polyhydroxylakanoate (PHA), a bacterial energy storage polymer. These tools were then used to characterize the responses of MTB to different O_2_ concentrations and iron-sufficient or iron-limited growth. Rapid analysis of MTB physiology will allow development of bioprocesses for the production of magnetosomes, and will increase understanding of this fascinating and useful group of bacteria.

## Introduction

Magnetic nanomaterials are increasingly important products with myriad applications in diverse settings including but not limited to environmental pollution control, information and energy storage^1^, catalysis^2^, biotechnological^1,3,4^ and especially biomedical research^1,5–7^. While most are produced by chemical means there is growing interest in harnessing the cellular machinery of certain naturally occurring bacteria^8–12^ to generate useful magnetic, and other metallic nanoparticle materials, biologically. In this context, ‘magnetosomes’, magnetic nanoparticle based organelles naturally contained within magnetotactic bacteria (MTB), are particularly important^8,13–17^. In most MTB, magnetosomes are arranged in one or more highly ordered ‘compass needle-like’ chains of single-domain permanently ferrimagnetic magnetite (Fe_3_O_4_) or greigite (Fe_3_S_4_) crystals (35–120 nm diameter) each wrapped in a phospholipid bilayer membrane containing a unique set of magnetosome specific proteins, i.e. distinct from those of cytoplasmic and outer membranes^8,13,15^. These internal structures within MTB function as navigational devices essential for magnetotaxis^18^. Unique properties of magnetosomes, not normally associated with chemically synthesized magnetic nanoparticles, of narrow size distribution, uniform morphology, high crystal purity, permanent magnetic character, high heating capacity, low aggregation tendency, ready dispersion in aqueous solution, facile functionalization, high biocompatibility, low toxicity and high specific absorption rates^10,12^ make them especially attractive prospects for biotech and healthcare applications, i.e. in immunoassays^19^, magnetic affinity cell sorting^20^, magnetic resonance imaging^21^, drug and gene delivery^22^ and cancer therapy^12,23^.

It is recognised that future widespread application of magnetosomes will, to a large extent, depend on the development of intensified high yielding manufacturing platforms for magnetosomes^10,12,16^. Fundamental to this are appropriate means for analysing MTB growth, viability, physiology and biomineralization of magnetic iron minerals, in order to understand and optimise magnetosome formation at any scale, from initial small (millilitre) studies on strain isolation and cultivability in the laboratory, and pilot scale manufacture (10–100 L), to fully fledged commercial production in cubic metre scale bioreactors. Qualitative evidence of magnetosome production within MTB can be obtained by observing a shimmering effect in cell suspensions mounted on magnetic stirrer plates, and black coloration of cell suspensions and/or colonies on agar plates, while magneto-spectrophotometric assay of cellular magnetism (C_mag_) of suspended cells provides a rapid indirect measure of cellular magnetosome content^24,25^. Quantitative determination of magnetosome content in cells and during subsequent recovery and purification operations usually involves measurement of iron content by means of atomic absorption spectrometry (dependent on species and cultivation conditions magnetosomes account for 80 to 99.5% of the total cell-bound iron in magnetic cells^18,26,27^), combined with imaging of magnetosomes by transmission electron microscopy. Recent work indicates the importance of monitoring physiological stress indicators to identify optimal conditions for magnetosome formation^28^, and the utility of transcriptome analysis for comparing magnetosome forming and non-forming conditions in MTB^29^. Other analytical methods especially pertinent to pilot- and large-scale magnetosome production and downstream processing from high biomass MTB fermentations include the tracking of polyhydroxyalkanoate (PHA) granules. Here, PHA formation diverts cellular resources from growth, lowering yields, and high levels of PHA would be likely to be a troublesome contaminant of magnetosome preparations. Current procedures for the determination of PHA content employ lengthy procedures involving solvent extraction, derivatization and gas chromatography^29^.

With the exception of at line optical density and C_mag_ measurements all of the aforementioned techniques are labour intensive and/or time consuming. The development of analytical methods is essential for the development of robust production processes, itself a requirement for industrialisation implementation. It is desirable that such methods will be rapid, requiring small volumes of samples and provide data of cellular parameters without the need of further growth, thus giving a ‘snapshot’ of the current physiological state of the cells. The flow cytometry (FCM) methods applied in this study fulfil these requirements. FCM has previously been applied previously for rapid analysis of microbial physiology^30^ and expression of auto-fluorescent proteins^31^, monitoring recombinant protein production^32^ and for investigating population heterogeneities in cultures. In FCM, multiple physical characteristics of single particles suspended in a fluid can be measured concurrently as they flow through a beam of light. FCM is a fast single-cell analysis technique well suited to collection of large datasets (tens of thousands of cells can be analysed) and allows determination of light scatter (relative size and granularity/internal complexity) and fluorescence properties of individual cells and thus determination of population heterogeneity. An important advantage of FCM is that it does not rely upon microbial growth for analysis of cell viability. ‘Viable but non-culturable’ (VBNC) cells exist within most microbial cell populations^33^, but growth-based methods for determining viable cell numbers (total viable counts generating colony forming unit, CFU data) will not detect the VBNC phenotype, thus total viable cell concentrations are underestimated. FCM does not share this limitation. MTB grow very slowly on agar plates, for example, *M. gryphiswaldense* MSR-1 forms colonies after 7–10 days^26^. Regardless of cell type FCM analysis can be performed in a matter of seconds. Moreover, when combined with carefully selected mixtures of fluorescent probes FCM can be employed to determine the physiological state of single cells.

Reports on the application of FCM to MTB are few in number^34,35^ and the full power of the technique has not exploited in any case. FCM has been used for analysing gene expression in *M. gryphiswaldense* MSR-1^34^, and in the development of new expression systems for the same species^35^. Green fluorescent protein (GFP) was used as a reporter in both studies, i.e. for magnetosomal localization and expression of GFP tagged magnetosome proteins under magnetite forming conditions^34^; and for identification of promoters (fused to GFP) for efficient gene expression^35^.

In this work, we present a battery of FCM methods tailored *a priori* to the study of *M. gryphiswaldense* MSR-1 and other MTB, and applicable to cells grown in liquid cultures and on agar plates. Specifically, we describe methods for determination of cellular concentration, cell size distribution, single-cell physiology and relative changes over time of intracellular contents of PHA and the chelatable iron pool.

## Results and Discussion

### Morphological difference between cells grown on plates and in suspension

**Figure 1:**
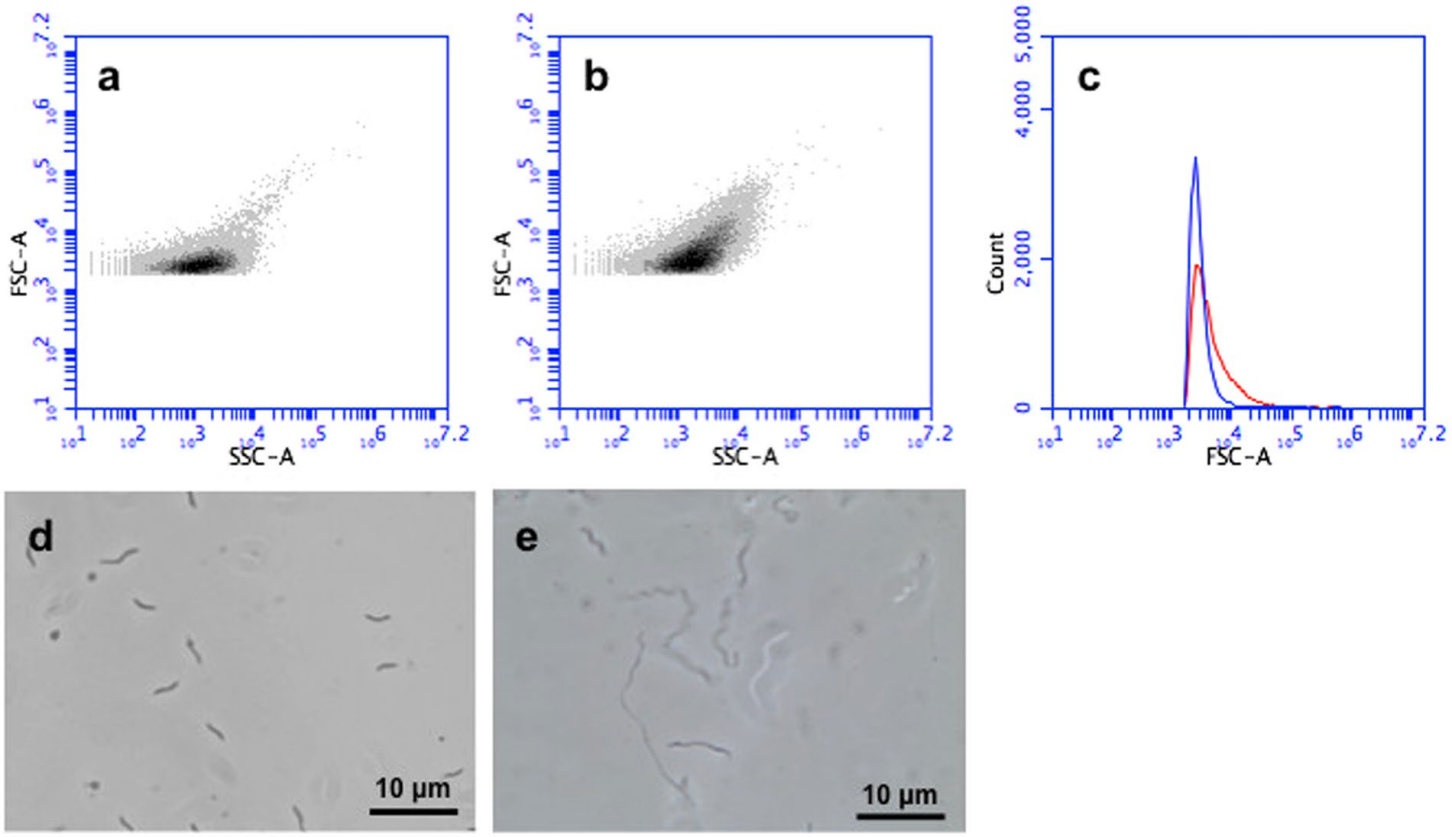
Analysis of *M. gryphiswaldense* MSR-1 using FSM and light microscopy. Scatter plots (Forward scatter, FSC-A vs. Side scatter, SSC-A) of cells cultured (**a**) in liquid FSM and (**b**) on ACA plates; (**c**) comparison of individual particle count vs FSC-A plots for liquid (red trace) and plate (blue trace) grown cells; light microscope images of (**d**) liquid and (**e**) plate grown cells.

FCM analysis was employed to monitor cell size and optical complexity of *M. gryphiswaldense* MSR-1 by means of light scattering. In FCM, light scatter is collected at two different angles: in the direction of the laser path (forward light scatter, FSC); and orthogonal to it (side scatter, SSC). For spherical particles (e.g. of latex), FSC correlates with the logarithm of particle diameter^36^. For cells and other non-spherical particles, changes in FSC are roughly indicative of changes in cell size. When applied to cells, SSC measures ‘granularity’^37^, a parameter that includes optical complexity caused by particulate material contained within the cell. Figure 1 shows the results of comparative FCM scatter and light microscopic analyses of MSR-1 cells cultured on ACA plates (resuspended in PBS) and in the liquid medium FSM. Clear differences in the heterogeneity of cell populations cultured in FSM (Fig. 1a) *cf*. those grown on ACA (Fig. 1b) can be discerned from the scatter patterns of FSC vs. SSC dot plots (Fig. 1a and b). Larger cells are represented by high FSC-A values (y-axis) whereas more granular cells are characterized by higher SSC-A (x-axis) values. Cells grown in liquid FSM appear less heterogeneous, smaller and less granular than those grown on ACA plates. Moreover, differences in particle size distribution and cell shape of suspension and plate grown MSR-1 cells are respectively inferred from ‘Count vs FSC-A’ histograms and light microscopy, with plate-grown cells appearing more polydisperse in size (Fig. 1c) and filamentous (Fig. 1e) compared to liquid-grown cells (Fig. 1d).

### Determination of cell concentration by FCM

FCM analysis was also used as a rapid method for determining cell concentrations in shake flask experiments. In auto-calibration mode and operating at a medium flow rate of 35 μL·min^−1^ a strong correlation (R^2^ > 0.95) between OD_565_ and FCM event counts was observed for MSR-1 cells (Supplemental Fig. S1a) with 1 OD_565_ equivalent to 1.16 × 10^9^ cells mL^−1^. This relationship is strikingly different to Schultheiss and Schüler’s^38^ correlation of OD_565_ with CFU, i.e. 1 OD_565_ equivalent to 3.3 × 10^8^ CFU mL^−1^ and likely reflects an important advantage of FCM over CFU counting, namely its ability to detect viable but non-culturable cells (VBNC). We also used Syto^**®**^62, a permeant DNA dye, to stain MSR-1 cells and so distinguish them from noise particles of similar size; with Syto^**®**^62-stained cells a similar correlation was found between OD_565_ and cell count (OD_565_ = 1.03 × 10^9^ cells mL^−1^; Supplemental Fig. S1b).

### Use of FCM to determine MSR-1 membrane polarization and cellular death

**Figure 2:**
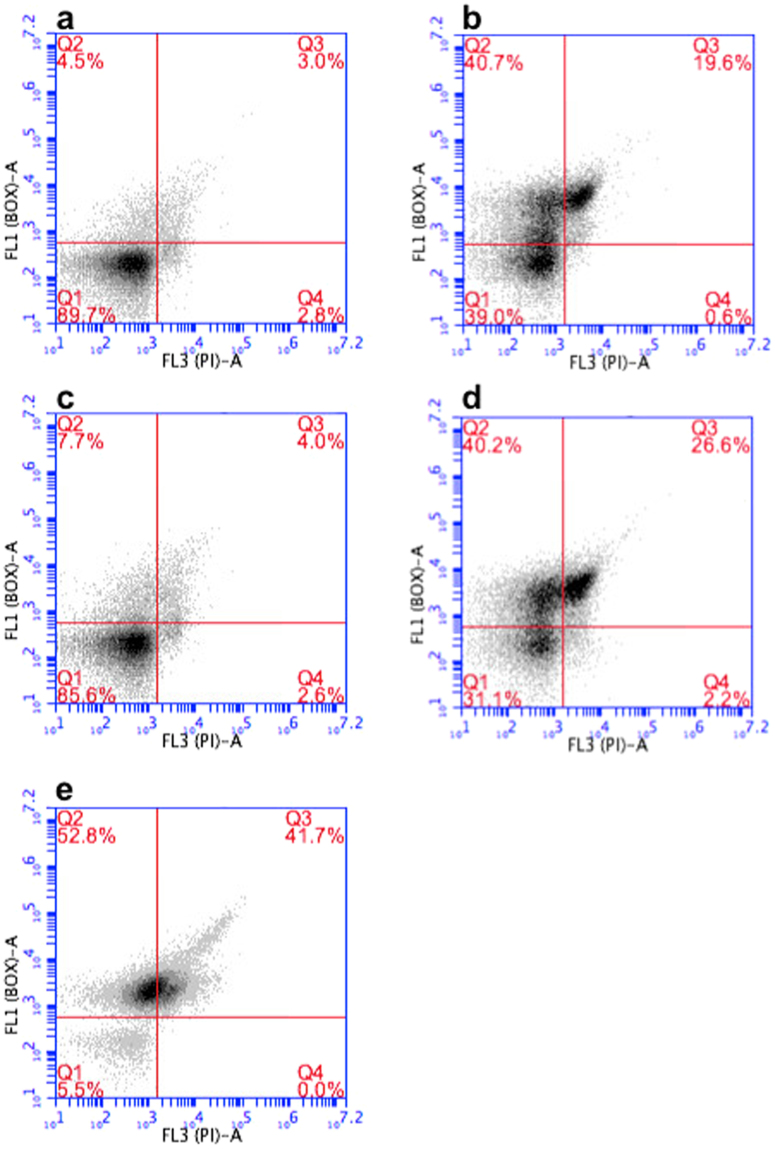
Viability analysis of MSR-1 cells using FCM. MSR-1 cells were co-stained with BOX (fluorescence measured on FL1-A channel, y axis) and PI (fluorescence measured on FL3-A channel, x axis). Key: (**a**) actively growing magnetic cells; (**b**) starving magnetic cells; (**c**) actively growing non-magnetic cells; (**d**) starving non-magnetic cells; and (**e**) cells incubated with absolute ethanol for 10 minutes, centrifuged and then resuspended in phosphate buffered saline. The numbers of cells in each of the four quadrants of all plots are indicated in red font and are expressed as percentages of the total population.

Two fluorescent probes were used to monitor the respiratory potential and death of *M. gryphiswaldense* MSR-1 cells using FCM. BOX (DiBac_4_^3^; bis-(1,3-dibutylbarbituric acid) trimethine oxonol) is a green lipophilic fluorescent probe that can only enter cells if their membranes are depolarized^39^. Healthy cells possess intact polarized cytoplasmic membranes, which are impermeant to BOX (BOX^−^). In contrast, cells with depolarised cytoplasmic membranes (whether injured, stressed or dead) permit BOX access and fluoresce green (BOX^+^). PI (propidium iodide), a nucleic acid intercalator, is excluded by the intact membrane of viable cells (PI^−^), but is taken up by dead cells which fluoresce red (PI^+^)^39^. Staining procedures were optimised using actively growing *M. gryphiswaldense* cells, starving cells and dead cells killed with ethanol. Figure 2 shows the two-colour fluorescence dot plots of MSR-1 cells co-stained with BOX and PI (fluorescence being detected on FL1-A and FL3-A channels, respectively). The fluorescence patterns from actively growing magnetic cells (Fig. 2a) and non-magnetic cells (Fig. 2c) were strikingly alike; i.e.: 86–90% of the cell populations were ‘healthy’, staining negatively with both fluorescent markers (BOX^−^ PI^−^, Quadrant 1); 5–8% were ‘injured’, staining positively with BOX, but negatively with PI (BOX^+^ PI^−^, Quadrant 2); and 3–4% were dead (BOX^+^ PI^+^, Quadrant 3). The fluorescence patterns from starving magnetic (Fig. 2b) and non-magnetic (Fig. 2d) MSR-1 cells were comparable with one another, but indicated noticeably fewer healthy populations (31–39% healthy, ~40% injured, 19–26% dead) than those of actively growing cells (~90% healthy, 5–8% injured, <3% dead). The low healthy population in starving cultures suggests the presence of large numbers of VBNC cells. This confirms the observation that the correlation between OD_565_ and cells·mL^−1^ as measured by FCM is different to the correlation between OD_565_ and CFU mL^−1^ ^38^ due to the presence of VBNC cells. Only 5% of the positive control population, i.e. cells killed with ethanol, were healthy MSR-1 cells (Fig. 2e).

### Accumulation of PHA aggregates in MSR-1

**Figure 3:**
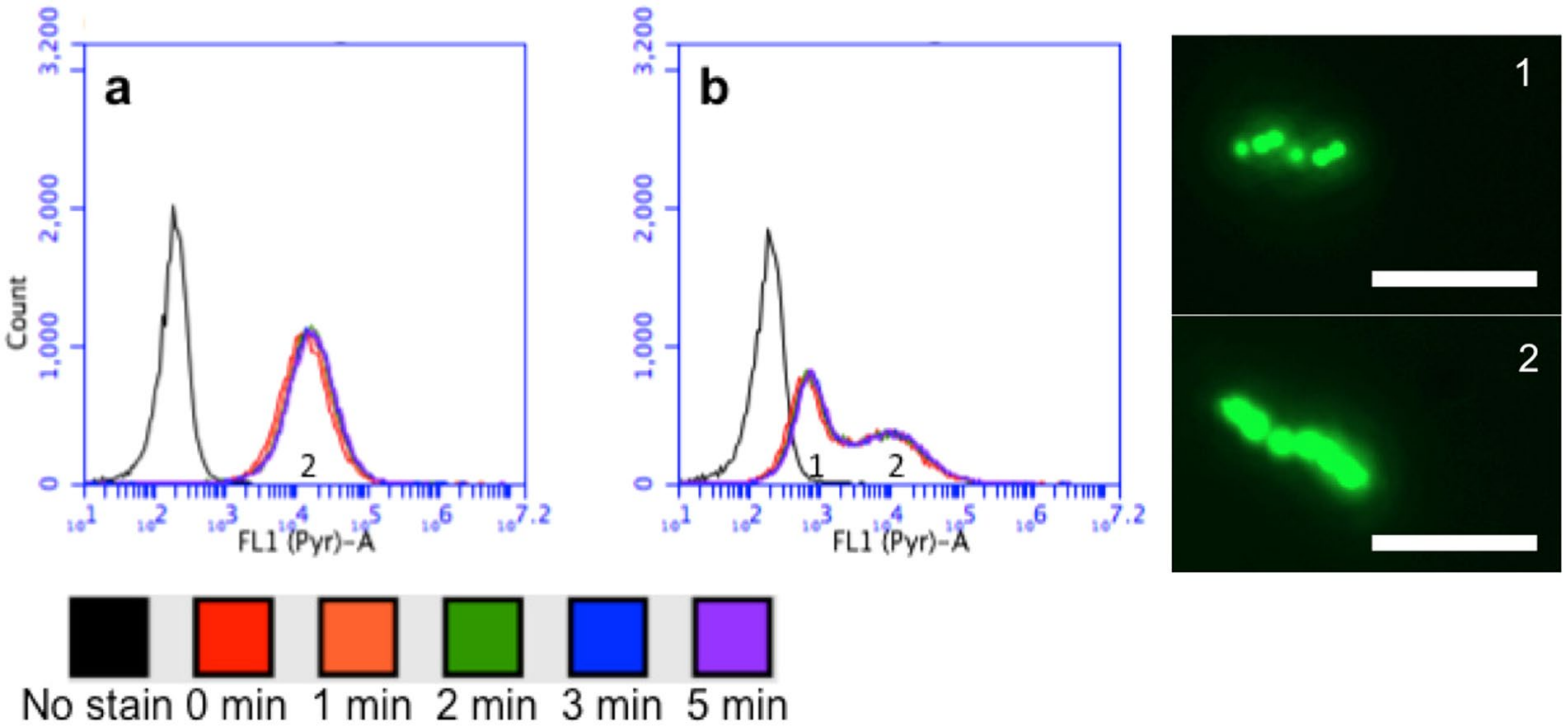
Analysis of PHA content using FCM. Fluorescence intensity histograms of starved (**a**) non-magnetic and (**b**) magnetic cells after staining with Pyr-546 (0.5 μg·mL^−1^) for various times. The numbers ‘1’ & ‘2’ marked on the inserts and the fluorescence micrographs correspond to non-magnetic and magnetic cells, respectively to identify those with low and high PHA content. The scale bars indicate a length of 5 μm.

It has been widely reported that limiting nitrogen and O_2_ availability under carbon excess results in high-level accumulation of polyhydroxyalkanoates (PHA) in several species of bacteria^40–44^. Ban *et al*.^43^ specifically examined the effect of hydrogen metabolism on cell growth and magnetosome synthesis in *M. gryphiswaldense* MSR-1 concluding that in MTBs PHA formation occurs under conditions of excess reducing power. Liu and co-workers^45^ succeeded in isolating an MSR-1 mutant capable of higher level magnetosome production and lower PHA accumulation than the wild type, indicating a possible link between the formation of PHA and magnetosomes in this bacterium. In more recent work, genomic excision of the *phbCAB* operon in MSR-1 was shown to abolish PHA granule formation albeit at the expense of much reduced growth^46^. Collectively, the above findings hint at the existence of an energy competition between the processes of PHA and magnetosome formation. Here we have used FCM to investigate PHA accumulation within individual bacteria of starved non-magnetic and magnetic MTB cultures. Cells were stained with the lipophilic dye 1,3,5,7,8-pentamethylpyrromethene-difluoroborate complex (pyrromethene-546 or Pyr-546) which on entering bacteria stains PHA green^47^. Previous studies have shown that Pyr-546 fluorescence correlates to intracellular PHA content^48^ and is superior to Nile red as a dye for PHA. After incubating for various times (10–300 s) samples were immediately analysed by FCM. Figure 3 shows that when used at a concentration of 0.5 μg·mL^−1^ the timeframe for Pyr-546 penetration and near full staining of intracellular PHA was the time taken to add the dye and analyse the sample (of the order of 10–15 s). No further enhancement in fluorescence occurred between 70 and 300 s exposure to Pyr-546 for both non-magnetic (Fig. 3a) and magnetic (Fig. 3b) cells. This said, FCM analysis reveals salient differences in the PHA content of magnetic and non-magnetic MSR-1, for example, revealing the presence of two discrete populations with low (Fig. 3b, labelled ‘1’) and high (Fig. 3b, labelled ‘2’) PHA content in magnetic cells *cf*. just a single ‘high PHA’ population in non-magnetic cells (Fig. 3a marked ‘2’). Fluorescence microscopy images of cells containing different quantities of Pyr-546 stained PHA are shown in Fig. 3. Similarly to our findings, recent studies performed with *Cupriavidus necator* observed sub-populations with more and less PHA^47^. Other works with *Pseudomonas putida* have recently observed an asymmetric PHA distribution during cell division under carbon limiting conditions suggesting that this could be explained by different cellular growth rates, distinct ability to degrade PHA or uneven distribution of PHA granules to daughter cells^49^.

### Measurement of intracellular chelatable iron

The intracellular pool of chelatable iron is considered a critical component in the biomineralization of magnetosomes. Recent studies in *M. gryphiswaldense* MSR-1 suggest that at least some of the iron transport for magnetite synthesis occurs through two copies of the ferrous iron transporter FeoB which is common to most bacteria. Strains lacking *feoB1* ^50^ and *feoB2* ^51^ were found to have lower magnetite contents than the wild type. Deletion of the iron response regulator, Fur, which activates *feoB1* and *feoB2* also resulted in reduced magnetosome formation^52^. All the above studies compare magnetosome production of wild type and ‘deficient’ strains, but do not provide dynamic measurements of iron transport in MSR-1. Moreover, it is well known that biomineralization depends not only on iron, but also on O_2_ availability^26,53^.

Typically, magnetosome production is quantified off-line (by measuring the iron content in cells using atomic absorption spectroscopy), and is backed up by visualization of magnetosomes under the transmission electron microscope. In both cases sample preparation and analysis are laborious and time consuming. Therefore there is clearly need of rapid new methods to interrogate and quantify magnetosome production and biomineralization in MTBs, as well as inform the development and optimization of large-scale magnetosome production strategies in bioreactors. It is this context that we developed a FCM based method for detecting chelatable iron in *M. gryphiswaldense* MSR-1 cells using phen green™ SK (PG-SK), whose fluorescence is quenched by metal ions including Fe^2+^ and Fe^3+^. PG-SK has been previously used to study iron transporters^54^ and efflux systems in *E. coli* as well as applied to studies in human cell lines for iron, copper and silver uptake^55,56^.

**Figure 4:**
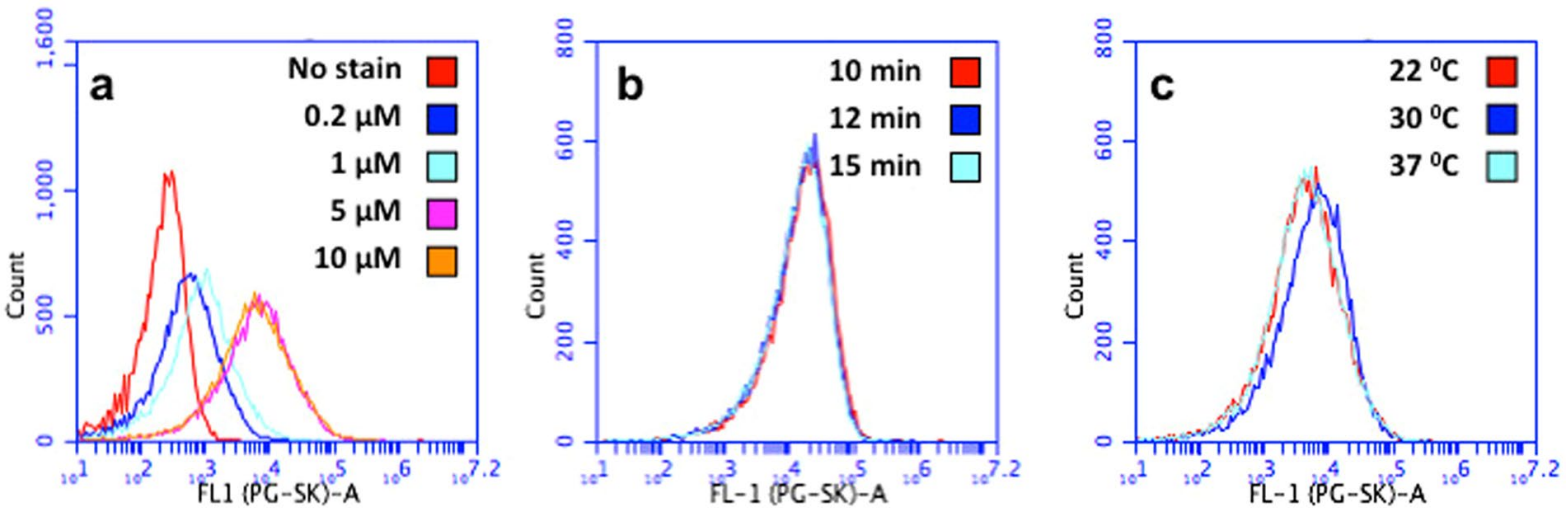
Analysis of intracellular iron by FCM. Fluorescence intensity histograms of non-magnetic MSR-1 cells growth in FSM-Fe^−^ after staining: (**a**) with various concentrations of PG-SK for 600 s at 30 °C; (**b**) for various times with 10 μM PG-SK at 30 °C; and (**c**) at various temperatures using 10 μM PG-SK for 600 s.

Non-magnetic MSR-1 cells grown aerobically in FSM-Fe^−^ (without iron) were stained with PG-SK. The staining procedure was partially optimized with respect to staining time (600–900 s) and PG-SK concentration (0.2–10 μM) at three different temperatures (22, 27 and 30 °C). The highest fluorescence was observed at a PG-SK concentration of 5 μM after 10 minutes of incubation (Fig. 4a). Doubling the PG-SK concentration did not enhance the fluorescence intensity of stained MSR-1 cells further (Fig. 4a), and longer staining times were not needed (Fig. 4b). Peak fluorescence intensity was similar at all staining temperatures employed (Fig. 4c).

### Physiological changes of cells cultured with limited O_2_ availability

**Figure 5:**
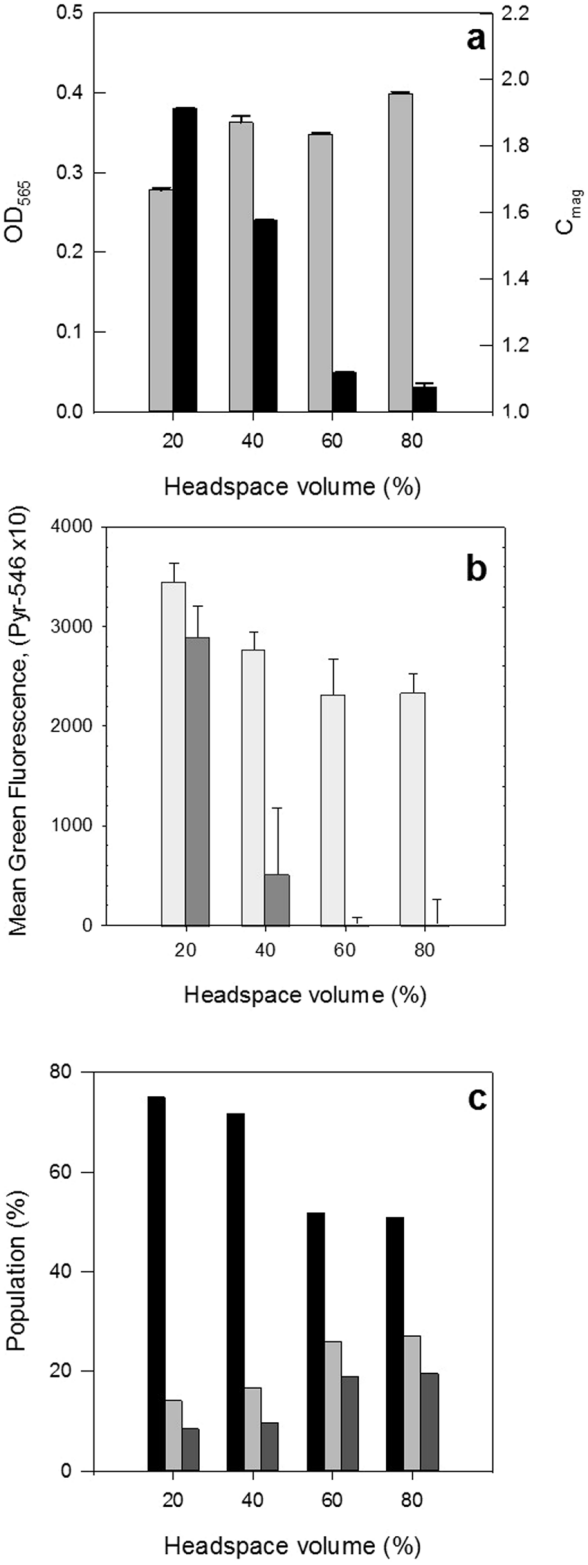
Effect of O_2_ limitation on physiology. MSR-1 cultures were grown in tubes with different headspace volumes for 48 h. (**a**) OD_565_ (pale grey bars) and cellular magnetism (C_mag_; black bars). Error bars are standard deviation. (**b**) Fluorescence of cells stained with PG-SK (dark grey bars) and Pyr-546 (white bars) as measured using FCM. Error bars are covariance. (**c**) Viability as determined using FCM and staining with PI and BOX; percentage of healthy (black bars), injured (pale grey bars) and dead (dark grey bars) cells are shown. Experiments were performed in triplicate.

The effect of O_2_ limitation on growth and cellular magnetism of MSR-1 cells was investigated indirectly by varying the volume of headspace provided (i.e. 20%, 40%, 60%, and 80%) in tightly sealed 50 mL Falcon tubes. In all experiments the initial OD_565_ was 0.086 ± 0.004. After 48 h in culture, OD_565_ and C_mag_ values were recorded (Fig. 5a). Two clear and opposite trends were observed; whereas biomass production paralleled the increase in headspace volume, and therefore O_2_ availability, conversely, the magnetism of *M. gryphiswaldense* MSR-1 cells dropped dramatically from strongly magnetic (C_mag_ = 2) at 20% (v/v) headspace to very weakly magnetic C_mag_ = 1.1 at 60% (v/v) headspace. These results are in agreement with findings from previous studies^26^. Figure 5b shows corresponding FCM analyses for relative quantification of intracellular iron and PHA content as a function of headspace volume. The highest intracellular PHA accumulation was observed in cells cultured in tubes with the lowest O_2_ availability (i.e. lowest headspace volume of 20%). Increased PHA formation during O_2_ limitation has previously been reported (reviewed by 41). Conversely, cells cultured at high O_2_ concentrations (80% headspace volume) had the lowest PG-SK fluorescence among the tested conditions and thus the highest free iron concentration. Microarray data showed that iron transporter *feoB1* is upregulated aerobically^29^, suggesting that iron transport into cells is highest aerobically.

Staining cells grown with different headspace volumes with PI and BOX (Fig. 5c) revealed that overall cell health was highest with 20% headspace and lowest with 80% headspace volume. PI was also used for analysis of physiology of MSR-1 cells from ACA agar plates; this indicated that 15–20% of cells on plates are dead (PI^+^). This highlights the difficulty in transferring MSR-1 cultures from single colonies to liquid cultures and emphasises the need to use a large amount of cells for setting up liquid cultures.

### Physiological characterization of MSR-1 in shake flask experiments with free air exchange

**Figure 6:**
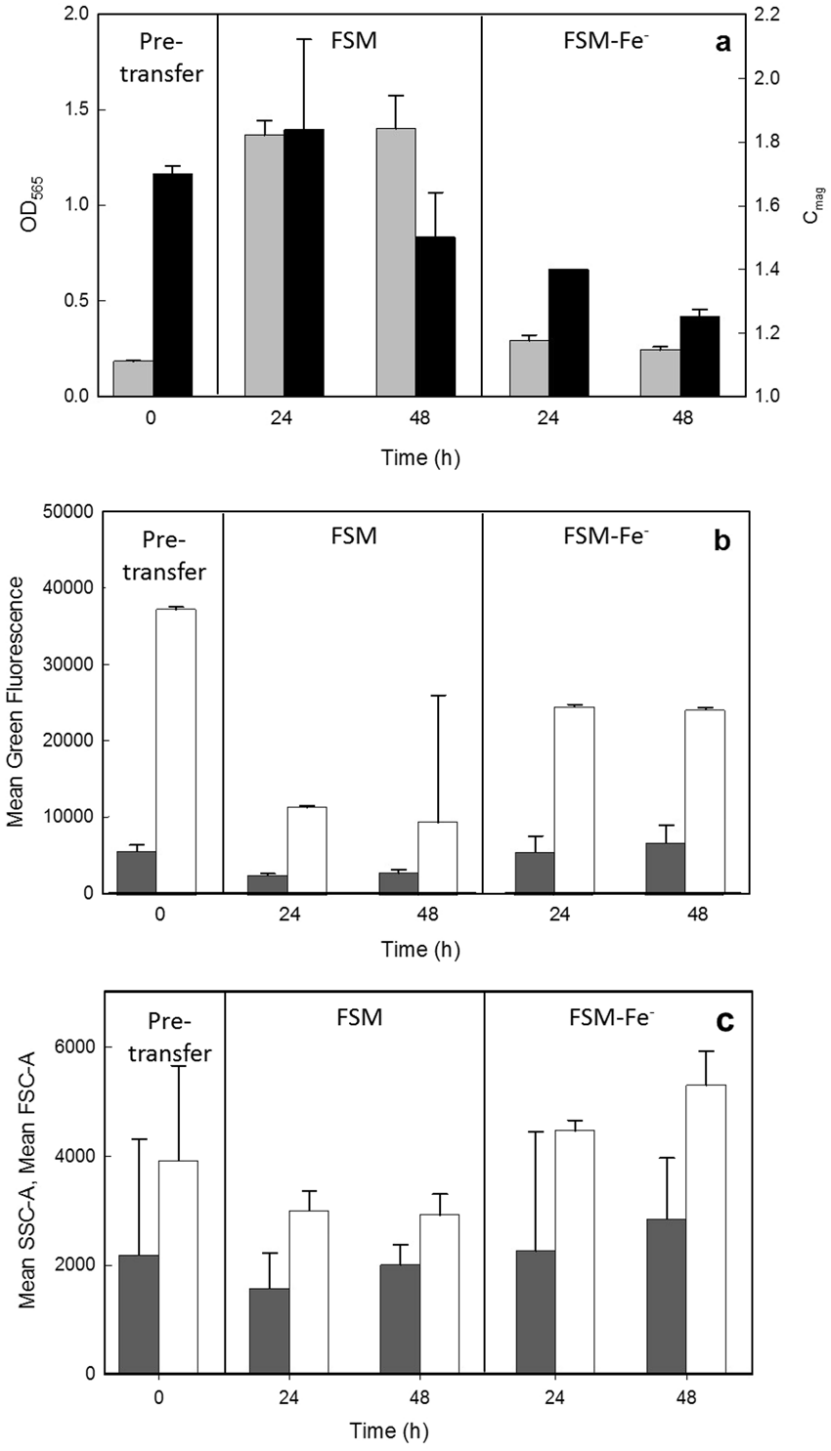
Physiology of magnetic cells during shift to aerobic conditions. MSR-1 cells grown under O_2_-limited conditions in a bioreactor were transferred to O_2_-sufficient conditions with either iron-containing (FSM) or iron-lacking (FSM-Fe^−^) media. (**a**) OD_565_ (pale grey bars) and C_mag_ value (black bars). Error bars are standard deviation; cells were taken from a single bioreactor into three replicate flasks for each condition. (**b**) Mean fluorescence intensity of cells stained with 0.5 μg·mL^−1^ pyrromethene-546 (Pyr546) (white bars) or 5 μM phen green™ SK (PG-SK) (dark grey bars). Error bars are covariance. (**c**) Forward scatter (FSC, white bars) and side scatter (SSC, grey bars) of cells as determined by FCM. Error bars are covariance. 25 000 events were analysed per sample by FCM.

To gain new insight into *M. gryphiswaldense* MSR-1 physiology during the shift from O_2_-limited to aerobic conditions, we transferred cells grown under O_2_-limited conditions to O_2_-rich conditions with or without the supplementation of iron. Magnetic cells grown in FSM batch medium and using a pH-stat feeding strategy in an O_2_-limited bioreactor were aseptically transferred to non-baffled shake flasks containing fresh media, either FSM or FSM without Fe (FSM-Fe^−^), and grown in O_2_ rich conditions (free air exchange) at 30 °C on an orbital shaker (150 rpm). OD_565_ and C_mag_ were monitored immediately before (t = 0 h) and 24 or 48 h after transfer and intracellular free iron and cellular PHA content were measured and compared to the pre-transfer culture using FCM (Fig. 6). After O_2_-limited growth in the bioreactor, and at the point of transfer to shake flasks (t = 0 h), MSR-1 cells were moderately magnetic (C_mag_ = 1.71). Strong Pyr-546 fluorescence (Fig. 6b, 0 h) and electron and fluorescence microscopy (Supplemental Fig. S2) confirmed that cells contained large quantities of PHA. After transfer to aerobic conditions, cells grew better in the presence of iron (FSM) compared to the absence (FSM-Fe^−^). After 24 h, C_mag_ rose slightly from 1.71 to 1.84 (although variation was high at 24 h), but then dropped to 1.46 at 48 h. Cultures grown in FSM had >3 fold decreased Pyr-546 fluorescence at 24 h and 48 h, indicating a decrease in PHA content, suggesting that growth utilised PHA as a substrate; FSC and SSC values also dropped (Fig. 6c), indicative of decreasing cell size and potentially corroborating loss of PHA granules. The impact of PHA utilisation on cell morphology has previously been reported in *C. necator* ^57^ and *Pseudomonas oleovorans* ^58^. In addition, in a parallel experiment, TEM analysis and fluorescence microscopy with Pyr-546 stained cells allow comparison of PHA detection methods (Supplemental Fig. S3).

After transfer of magnetic cells to culture medium lacking iron citrate (FSM-Fe^−^), very little growth ensued (OD_565_ ~ 0.3; Fig. 6a). C_mag_ fell steadily (reaching 1.2 after 48 h; Fig. 6a). Pyr-546 fluorescence dropped to 65% of its pre-transfer value (Fig. 6b), reflecting low PHA utilisation, whereas FSC and SSC increased, indicating an increase in cell size and granularity (Fig. 6c).

PG-SK fluorescence dropped over time for cultures grown in FSM but not in the absence of iron; as expected, this reflects an increase in chelatable iron concentration in cells grown in the presence of iron, but not in the absence. Headspace volume experiments revealed an increase in intracellular iron concentration under more aerobic conditions in the presence of iron (Fig. 5). As with the regulation of intracellular iron concentrations in response to O_**2**_, high extracellular iron concentrations were shown to increase the expression of the *feo* iron transporters^59^.

In summary, FCM analysis of viability, intracellular chelatable iron and PHA, employing PI/BOX, PG-SK and Pyr-546 dyes respectively provides valuable insight on the effects of O_2_ and iron levels on the growth, magnetosome and PHA production of MTBs. The data are rapidly obtained, does not require growth of MTBs on agar plates, and when used together with similarly fast measurements of optical density and C_mag_ can be useful in the design of growth strategies for production of magnetosome rich cells.

## Methods

### Strains, growth media and culture conditions

*Magnetospirillum gryphiswaldense* MSR-1 was obtained from Leibniz-DSMZ (Deutsche Sammlung van Mikroorganismen und Zellkulturen GmbH) and used in all experiments. Unless otherwise indicated all chemicals were from Sigma-Aldrich Chemical Company Ltd (Gillingham, Dorset, UK). *M. gryphiswaldense* MSR-1 cells were grown on solid activated charcoal agar (ACA) plates and in liquid media. ACA plates contained 3 g L^−1^ activated charcoal. 15 g L^−1^ agar (Formedium, Hunstanton, Norfolk, UK), 0.1 g L^−1^ yeast extract, 3 g L^−1^ soybean peptone, 3 g L^−1^ sodium pyruvate, 0.34 g L^−1^ NaNO_3_, 0.1 g L^−1^ KH_2_PO_4_, 0.15 g L^−1^ MgSO_4_·7H_2_O and 2.38 g L^−1^ 4-(2-Hydroxyethyl)piperazine-1-ethanesulfonic acid (HEPES) buffer in MiliQ water. The pH was adjusted to 7.0 with sodium hydroxide (Heyen and Schüler, 2003) before autoclaving. After autoclaving iron(III) citrate (final concentration of 500 µM) and 1.4-dithiothreitol (DTT; final concentration of 1 mM) were aseptically added to the plate mix before pouring^38^. Set ACA plates were incubated at 30 °C in 12-plates anaerobic jars with one Anaerocult^®^C sachet (Merck Chemicals Ltd, Beeston Notts, UK) to achieve microaerobic conditions. Liquid cultures of *M. gryphiswaldense* MSR-1 were routinely grown in a shaking incubator (30 °C, 150 rpm) using a flask standard medium (FSM) composed of 0.1 g L^−1^ yeast extract, 3 g L^−1^ soybean peptone, 3.5 g L^−1^ potassium l-lactate, 100 µM iron(III) citrate, 0.34 g L^−1^ NaNO_3_, 0.1 g L^−1^ KH_2_PO_4_, 0.15 g L^−1^ MgSO_4_·7H_2_O, 5 mL L^−1^ of EDTA-chelated trace element mixture^60^ and 2.38 g L^−1^ HEPES buffer in deionized water; the whole adjusted to pH 7.0 prior to sterilization in an autoclave. Cells were grown at 30 °C in a shaking incubator at 150 rpm. O_2_-limiting cultures were grown in tightly sealed screw cap 50 mL Falcon tubes with variable headspace volumes (10–40 mL), whereas aerobic cultivations were performed with 50 mL of media in 250 mL shake flasks allowing free air exchange. Non-magnetic cells were cultured in FSM without iron (FSM-Fe^−^) for a minimum of three sequential sub-cultures in an attempt to eliminate all trace of the metal. Magnetic cells were obtained from cultures grown in bioreactor experiments under controlled conditions using a growth strategy adapted from previous works^26,28,61^.

### Flow cytometry (FCM)

Bacterial samples taken directly from agar plates or liquid cultures were resuspended in phosphate-buffered saline (PBS) and then analysed directly in a BD Accuri C6 flow cytometer (Becton, Dickinson and Company, Oxford, UK) for cell size and granularity, or after staining with various fluorescent dyes (see Supplemental Table 1) using protocols developed and detailed in the Results and Discussion. During FCM on fluorescently labelled cells, samples were excited using a 488 nm solid-state laser and fluorescence was detected using two different filters, i.e.: a 533/30 BP filter (FL1-A) for bis (1.3-dibutybarbituric acid) trimethine oxonol (referred to here as bis-oxonol or BOX), pyrromethene-546 (Pyr-546) and phen green™ SK (PG-SK); and a 670 LP filter (FL3-A) for propidium iodide (PI). Syto^®^62 was excited with a 640 nm solid-state laser and detected through a 675/25 BP filter (FL4-A).

### Analytical methods

Culture optical densities were recorded at a wavelength of 565 nm (OD_565_) in an Evolution 300 UV-Vis spectrophotometer (Thermo Fisher Scientific, Hemel Hempstead, Herts, UK) controlled by Thermo Scientific™ VISION*pro*™ software.

Cellular magnetic response (C_mag_) values of cultures were determined immediately after OD_565_ measurements using a purpose-built magnetic measurement system mounted within the spectrophotometer. In common with previous magneto-spectrophotometry apparatus^24,25^ our system features two pairs of Helmholtz coils arranged around the cuvette holder, one pair perpendicular to the light beam and the other in line with it. Each pair of coils is energized in turn, and the OD_565_ is measured in each condition. Magnetic cells will align with the magnetic field and thus orient in line with or perpendicular to the light beam; the optical density will therefore change between the two conditions. Non-magnetic cells do not align with the magnetic field, thus the optical density does not change on switching the field orientation. C_mag_ values for culture samples are calculated by dividing the OD_565_ values of suspensions of cells aligned parallel to the light beam by those obtained when the same cells are perpendicularly aligned. C_mag_ values greater than unity reflect the presence of magnetic cells.

Cellular morphology was routinely examined by light microscopy using an Olympus BX50 optical microscope (Olympus Corporation, Tokyo, Japan). Images were captured using a MotiCam 1 (800 × 600 pixel) camera (Microscope Systems Limited, Glasgow, UK) and processed with Motic Images Plus 2.0 software (Motic Europe S.L.U., Barcelona, Spain).

Cells stained with fluorescent probes were observed and imaged using a Zeiss Axiolab microscope (Carl Zeiss Ltd., Cambridge, UK) fitted with a Zeiss AxioCam ICm1 camera, and the images were processed in auto-exposure mode with the aid of Zeiss ZEN Lite 2012 software. Samples were excited with a Zeiss VHW 50f-2b ultraviolet light source and a 520 LP filter was employed for detection of fluorescence from Syto^**®**^ 9 and pyrromethene-546 (Pyr-546).

### Data availability

The datasets generated during and analysed during the current study are available from the corresponding author on reasonable request.

## Electronic supplementary material

Supplementary information
