# Supplementary material for: Flow cytometry as a rapid analytical tool to determine physiological responses to changing O2 and iron concentration by Magnetospirillum gryphiswaldense strain MSR-1
Source: Sci Rep. 2017 Oct 13;7:13118. doi: 10.1038/s41598-017-13414-z (PMC5640647; doi:10.1038/s41598-017-13414-z)
Supplement: Supplementary file 1 — Supplementary information [file 41598_2017_13414_MOESM1_ESM.pdf]

Supplemental information

Flow cytometry as a rapid analytical tool to determine physiological responses to changing O_2 and Fe concentration by *Magnetospirillum gryphiswaldense* strain MSR-1

Alfred Fernández-Castané,[a.b]† Hong Li,[a] Owen RT Thomas,[a] Tim W Overton*[a.b]

[a]School of Chemical Engineering. College of Engineering and Physical Sciences and [b]Institute for Microbiology and Infection. University of Birmingham. Edgbaston. Birmingham. B15 2TT. UK.

*to whom correspondence should be addressed: t.w.overton@bham.ac.uk, +44 (0) 121 414 5306

† Current address: School of Engineering and Applied Science, Aston University, Birmingham, B4 7ET, UK.

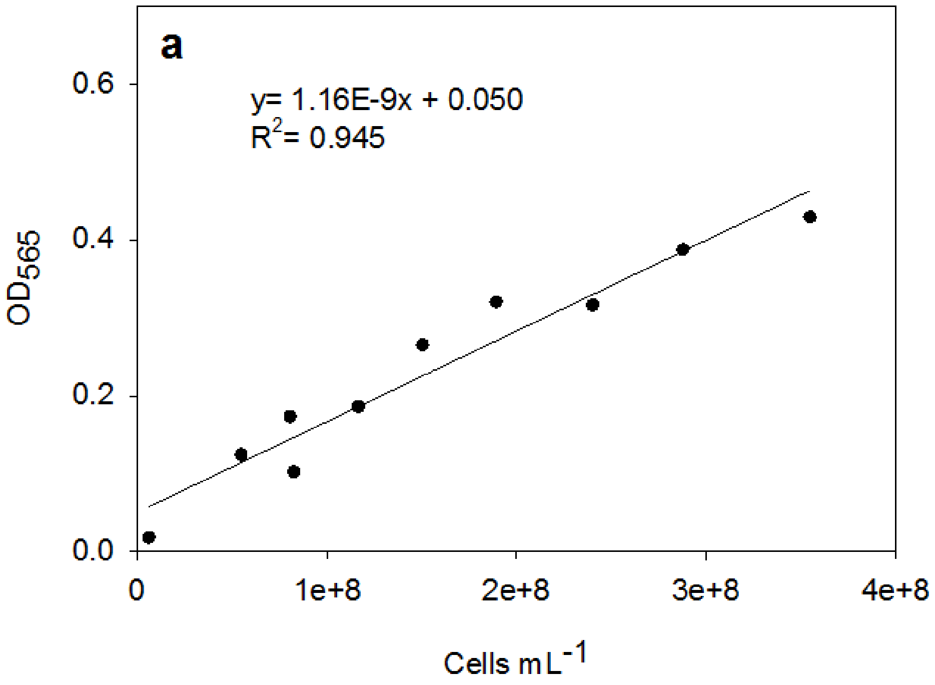

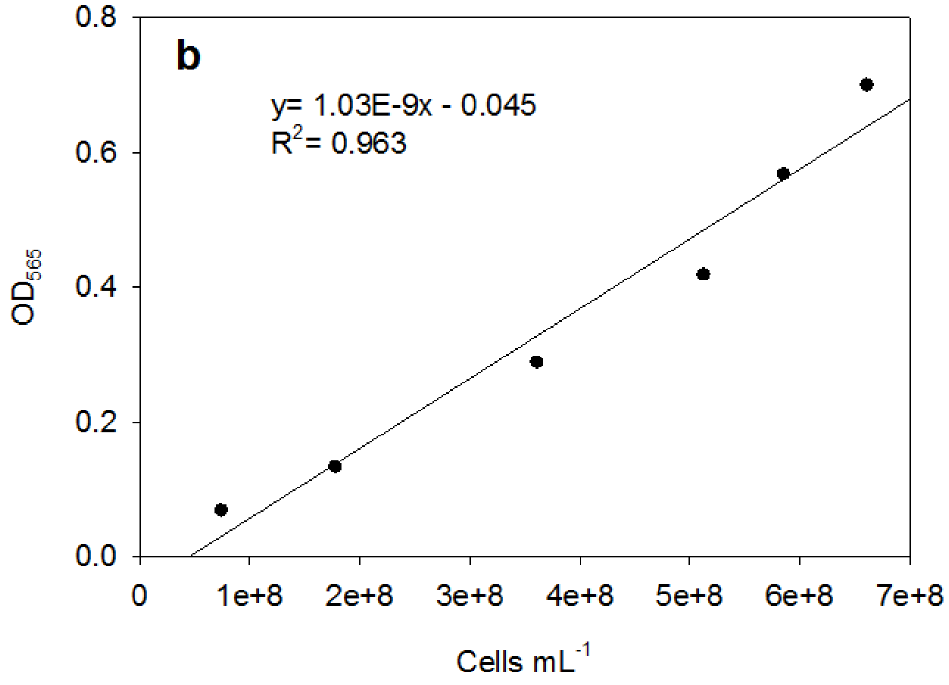

Supplemental Figure S1. Correlation between culture OD_{565} and cells·mL^{-1} measured using FCM. (a) Non-stained cells (b) cells stained with Syto62.

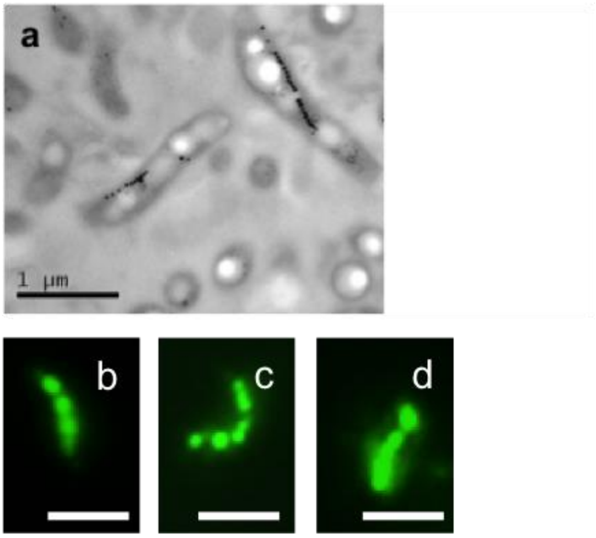

Supplemental Figure S2. (a) TEM micrograph of *M. gryphiswaldense* MSR-1 cells harvested from an O_2-limited bioreactor. Note the magnetosome chains (black) and PHA aggregates (white globules). Fluorescence images of Pyr-546 stained MSR-1 cells: (b) harvested from an O_2-limited bioreactor and after 48 h growth on (c) FSM and (d) FSM-Fe^{-} medium. The scale bars indicate a length of 2.5 µm.

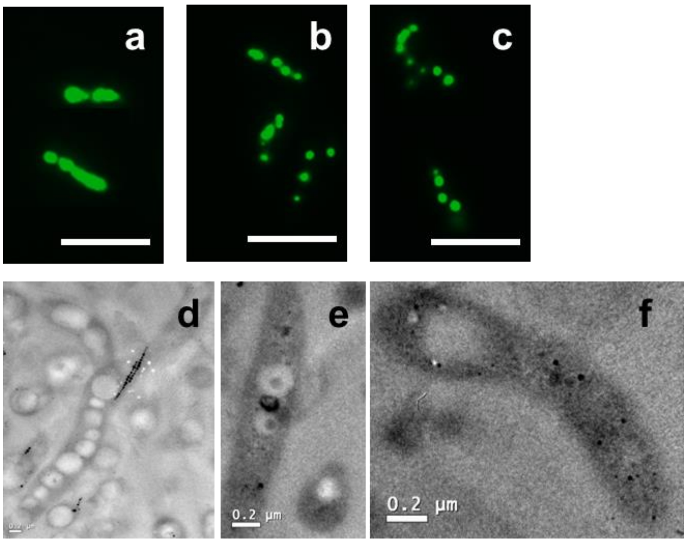

Figure S3. Comparison of fluorescence microscopy and TEM images of magnetic cells during shift to aerobic conditions. MSR-1 cells grown under O_2-limited conditions in a bioreactor were transferred to O_2-sufficient conditions with either iron-containing (FSM) or iron-lacking (FSM-Fe^-) media. Fluorescence images of Pyr-546 stained MSR-1 cells: (a) harvested from an O_2-limited bioreactor and after 48 h growth on (b) FSM and (c) FSM-Fe^- medium; the scale bars indicate a length of 5 µm. TEM micrographs of MSR-1 cells: (d) harvested from an O_2-limited bioreactor and after 48 h growth on (e) FSM and (f) FSM-Fe^- medium.

Supplemental Table 1. Fluorescent dyes used in this study. With the exception of Pyr-546 (Photonic Solutions, Ohio, USA) the listed dyes were acquired from Fisher Scientific (Loughborough, Leics, UK). The cited working concentrations were adapted from previous studies and/or optimised in this work.

Fluorescent dye	Excitation $\lambda_{max, abs}$ (nm)	Emission $\lambda_{max, fl}$ (nm)	Stock concentration	Solvent	Working concentration	Fluorescence channel
Bis-(1,3-dibutylbarbituric Acid) Trimethine Oxonol (BOX)	490	516	10 mg·mL^{-1}	DMSO	100 ng/mL	FL1 (green)
Phen Green™ SK (PG-SK)	525	580	1 mM	DMSO	5 µM	FL1 (green)
Propidium iodide (PI)	533	617	200 µg·mL^{-1}	H_2O	100 ng/mL	FL3 (red)
Pyrromethene-546 (Pyr-546)	493	519	0.1 mg·mL^{-1}	DMSO	0.5 µg/mL	FL1 (green)
Syto®9*	483	503	5 mM	DMSO	1 µM	N/A
Syto®62	652	676	5 mM	DMSO	0.4 µM	FL4 (far red)

*Syto® 9 was only used for fluorescence microscopy assays. N/A = not applicable.
